# Predictive model for converting optic neuritis to multiple sclerosis; decision tree in focus

**DOI:** 10.1371/journal.pone.0309702

**Published:** 2024-12-02

**Authors:** Saeid Rasouli, Mohammad Sedigh Dakkali, Azim Ghazvini, Reza Azarbad, Mahdi Asani, Zahra Mirzaasgari, Mohammed Arish

**Affiliations:** 1 Five Senses Health Research Institute, School of Medicine, Hazrat-e Rasool General Hospital, Iran University of Medical Sciences, Tehran, Iran; 2 Department of Ophthalmology, School of Medicine, Al Zahra Eye Hospital, Zahedan University of Medical Sciences, Zahedan, Iran; 3 School of Medicine, Iran University of Medical Sciences, Tehran, Iran; 4 Health Research Institute, Cellular and Molecular Biology Research Center, Babol University of Medical Sciences, Babol, Iran; 5 Department of Neurology, Firoozgar Hospital, School of Medicine, Iran University of Medical Science, Tehran, Iran; Karolinska Institutet, SWEDEN

## Abstract

**Background:**

Optic neuritis (ON) can be an initial clinical presentation of multiple sclerosis This study aims to provide a practical predictive model for identifying at-risk ON patients in developing MS.

**Method:**

We utilized data from the Optic Neuritis Treatment Trial study, which enrolled 457 patients aged from 18 to 46 years, all diagnosed with acute ON. These patients underwent up to 15 years of neurological and ophthalmologic examinations and imaging. The selection of variables for the developing model was based on clinical importance and statistical significance, and any missing values were appropriately addressed. We developed a Decision Tree (DT) classifier as the primary model and manually tuned its hyperparameters for optimal performance. We employed SHapley Additive exPlanations (SHAP) for feature importance evaluation. All analysis was performed using Python version 3.10.9 and its associated libraries.

**Results:**

A total of 388 patients completed the study, of which 154 developed clinically definite multiple sclerosis (CDMS). It was observed that 61% of patients with magnetic resonance imaging (MRI) lesions developed CDMS. The final variables selected for analysis were MRI lesions, neurologic history, ON type, gender, and visual field mean deviation. The DT model achieved an accuracy of 70.1% during cross-validation and 69.1% on the test set, with an area under the curve of 74.9% and 71.7%, respectively. Comparative analysis of DT with other models showed similar performance. SHAP analysis revealed that MRI lesions and ON type emerged as the two most significant features, with relative importance of 61% and 18%, respectively.

**Conclusion:**

The decision tree model, with satisfactory performance, effectively stratifies patients, based on baseline findings and offers valuable insights for informed decision-making by physicians.

## Introduction

Optic neuritis (ON), a neuroinflammatory condition, primarily affects the optic nerve, manifesting as unilateral, subacute symptoms, including ocular pain and visual impairment [1,2]. ON can present as an isolated disorder or as a symptom of an underlying condition, such as multiple sclerosis (MS) [3]. About 20% of MS patients had ON as their first clinical manifestation, and 50–65% of MS patients experienced ON during their disease [4,5]. Therefore, predicting the conversion of ON to clinically definite multiple sclerosis (CDMS) is crucial for determining effective factors, selecting at-risk patients for monitoring, follow-up, and initiating early treatment [1,6].

Previous research has identified factors such as, age, gender, and prior neuritis episodes, that are associated with the conversion of ON to MS [6,7]. Additionally, the presence of demyelinating plaques on magnetic resonance imaging (MRI) is a powerful predictor of developing MS [8]. A few models have attempted to predict this conversion; they vary in effectiveness and accessibility. For example, Luo et al. [9] developed a predictive model achieving a concordance index (C-index) of 0.72. In contrast, another model based on cerebrospinal fluid biomarkers reached an area under the curve (AUC) of 0.90 [10]. Rasouli et al. [11] presented a model for converting clinically isolated syndromes (including ON and transverse myelitis) to MS, demonstrating an accuracy of 83.6% and an AUC of 91.8%. Although these models are promising, two are web-based, and one relies on invasive procedures. Notably, only the study by Abri-Aghdam et al. [6] presented a decision tree for predicting MS development in ON patients, achieving an accuracy of 74%. Their classification tree model, focusing on patients with positive plaques on MRI, identified nine nodes, where the probability of MS conversion was estimated for each node.

Despite these advancements, there is still a critical gap in developing a predictive model that is accessible, interpretable, user-friendly, and suitable for integration into clinical settings. Our study aims to bridge this gap using machine learning techniques. While our primary focus is on the decision tree classifier, we have also employed other machine learning models to validate and ensure the robustness of the decision tree results. For this purpose, we utilized comprehensive data from the Optic Neuritis Treatment Trial (ONTT), which includes MRI results, demographic information, and clinical evaluations [4,7]. Through this approach, we hope to provide a practical algorithm for risk stratification and prediction of MS development in ON patients with or without plaque on MRI.

## Materials and methods

### Source of data

Patients were recruited from the ONTT database. From July 1, 1988, to June 30, 1991, the trial enrolled 457 patients, aged from 18 to 46 years, all diagnosed with unilateral acute ON [12,13]. Before enrollment in this study, there was no history of acute ON in the affected eye or systemic disease that can cause this condition, other than MS. Patients were randomly assigned to three treatment groups: low-dose oral prednisone, high-dose intravenous methylprednisolone (IVMP) followed by oral prednisone, and oral placebo. Standardized ophthalmic and neurological examinations were performed at enrollment, at 6 and 12 months, then annually for up to 5 years after registration and then at years 10 and 15. Standardized unenhanced brain MRI was performed at enrollment, and the number of white matter lesions of at least 3 mm in diameter on T2-weighted MRI was determined.

**Fig 1:**
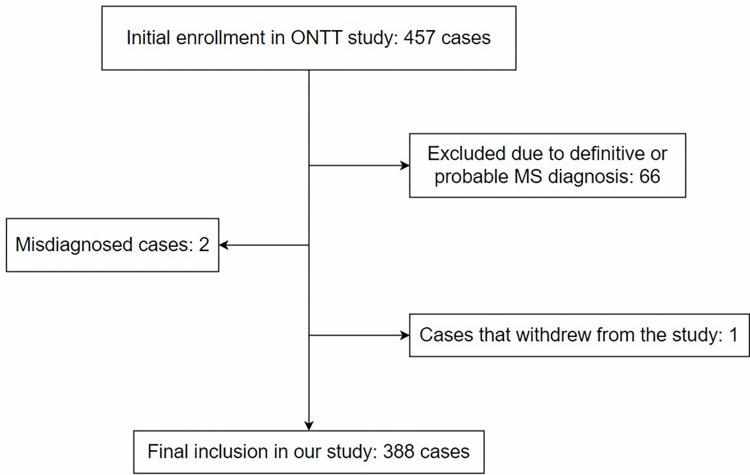
Flowchart of the study participants.

Diagnoses were derived from the ONTT by applying Poser’s criteria during the study follow-up period. The potential predictive variables associated with CDMS conversion were selected from the demographic and clinic-radiological features at the time of enrollment. These selections were based on the clinical importance, the physician’s professional opinion, and previously published evidence. Patients were followed up for their visual outcomes and the development of CDMS for 15 years. Out of the 457 participants initially enrolled in the trial, 391 were diagnosed with ON without a definitive or probable diagnosis of MS at the time of enrollment. However, after study entry, ON was ruled out in two patients, and one patient withdrew prior to undergoing a baseline neurologic examination (Fig 1). Several centers participated in the ONTT study, and each center received ethical approval from the investigational board. Written informed consent forms were obtained from each participant in the study and the trial registered at clinicaltrials.gov under the trial number NCT00000146. The source of the data is [ONTT Study Group. (2006). ONTT and Longitudinal Optic Neuritis Study (LONS), which includes follow-up through 15 years. NCT00000146]. Retrieved from https://public.jaeb.org/lons/stdy. The clean public dataset used in this study was accessed online at https://www.kaggle.com/datasets/rasoulisaeid/ontt-derived-data. The analyses, content, and conclusions presented herein are solely the responsibility of the authors and have not been approved by the ONTT Study Group.

### Variable selection

**Fig 2:**
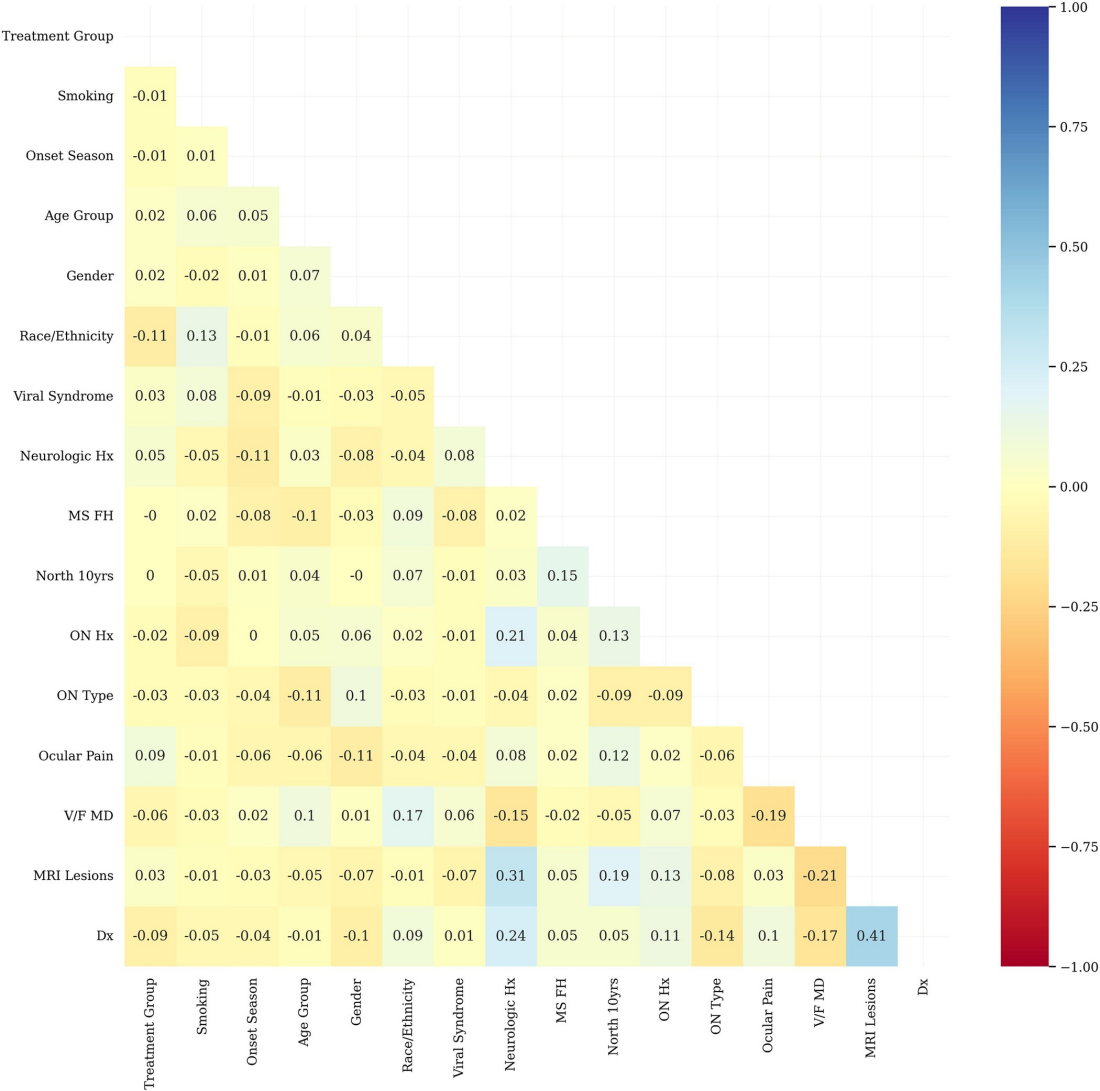
Correlation matrix of variables. Hx: History; MS: Multiple sclerosis; ON: Optic neuritis; VF MD: Visual field mean deviation; MRI: Magnetic resonance imaging; Dx: Diagnosis.

The dataset includes demographic information such as age and gender, as well as clinical data from ophthalmic examinations and MRI results. We used only the baseline data for our study, except for the final diagnosis and family history of MS, as these could change during the trial. Then, we computed a correlation matrix for all baseline variables. From the variables that exhibited more than 50% correlation with each other, we selected the one that had a higher correlation with the target variable among each set of correlated variables. Subsequently, we chose 15 variables for the final analysis (Table 1 and Fig 2).

**Table 1:** Clinical characteristics and diagnostic markers in CDMS and non-CDMS patients.

Variables	Overall (n = 388)	non-CDMS	CDMS	RR (95% CI)	p
Age group					0.90
< 30 years	161	96	65	1.03 (0.80, 1.32)	
≥ 30 years	227	138	89	0.97 (0.76, 1.24)	
Gender					0.05
Female	299	172	127	1.40 (1.00, 1.97)	
Male	89	62	27	0.71 (0.51, 1.00)	
Treatment group					
Placebo or Prednisone	255	146	109	1.26 (0.96, 1.67)	0.11
Intravenous	133	88	45	0.79 (0.60, 1.04)	
Smoking history					0.35
Negative	194	112	82	1.14 (0.89, 1.46)	
Positive	194	122	72	0.88 (0.69, 1.12)	
Onset season					0.50
Fall or Winter	187	109	78	1.10 (0.86, 1.41)	
Spring or Summer	201	125	76	0.91 (0.71, 1.16)	
VF mean deviation					0.003
> -6 dB	31	27	4	0.31 (0.12, 0.77)	
≤ -6 dB	255	205	150	3.27 (1.30, 8.24)	
Missed	2	2	0		
Race/ethnicity					0.10
Other	58	41	17	0.71 (0.46, 1.07)	
White	230	193	137	1.42 (0.93, 2.16)	
Viral syndrome					0.10
Negative	286	173	113	0.98 (0.75, 1.30)	
Positive	102	61	41	1.02 (0.77, 1.34)	
Neurologic history					<0.0001
Negative	323	212	111	0.52 (0.41, 0.65)	
Positive	65	22	43	1.93 (1.53, 2.42)	
MRI lesions					
Negative	191	152	39	0.34 (0.25, 0.46)	
Positive	160	63	97	2.97 (2.19, 4.03)	
Missed	37	19	18		
ON type					<0.009
Retrobulbar	240	132	108	1.45 (1.10, 1.91)	
Papillitis	148	102	46	0.69 (0.52, 0.91)	
Ocular pain					0.07
Negative	257	210	147	0.55 (0.28, 1.06)	
Positive	31	24	7	1.82 (0.94, 3.54)	
ON history					0.06
Negative	271	228	143	0.60 (0.41, 0.87)	
Positive	17	6	11	1.68 (1.16, 2.44)	
MS FH					0.37
Negative	241	209	132	0.83 (0.59, 1.15)	
Positive	47	25	22	1.21 (0.87, 1.69)	
North 10yrs					0.35
Negative	161	102	59	0.88 (0.68, 1.13)	
Positive	227	132	95	1.14 (0.89, 1.47)	

CDMS: Clinically definite multiple sclerosis; RR: Risk ratio; CI: Confidence interval; p: p-value; ON: Optic neuritis; FH: Family history; VF: Visual field; Oral: Oral cortisone; North 10yrs: Lived in the north 10 or more of the first 15 years of life (north is defined as states predominantly located above latitude 40 degrees north); Viral syndrome: Has the patient had viral syndrome within the previous month?

In our study, we transformed all variables into binary values. We applied specific rules to variables that were not initially binary. For age, we used 30 years as the threshold. In the treatment group, individuals who received intravenous treatment were distinguished from others. For the onset season, we differentiated between cases with onset in spring or summer and those with onset in fall or winter. For visual field mean deviation (MD), cases where the deviation was equal to or less than -6 dB were separated. For MRI lesions, patients with at least one lesion in the MRI were identified separately. This binarization process simplifies the application of machine learning algorithms and the interpretation of results.

**Fig 3:**
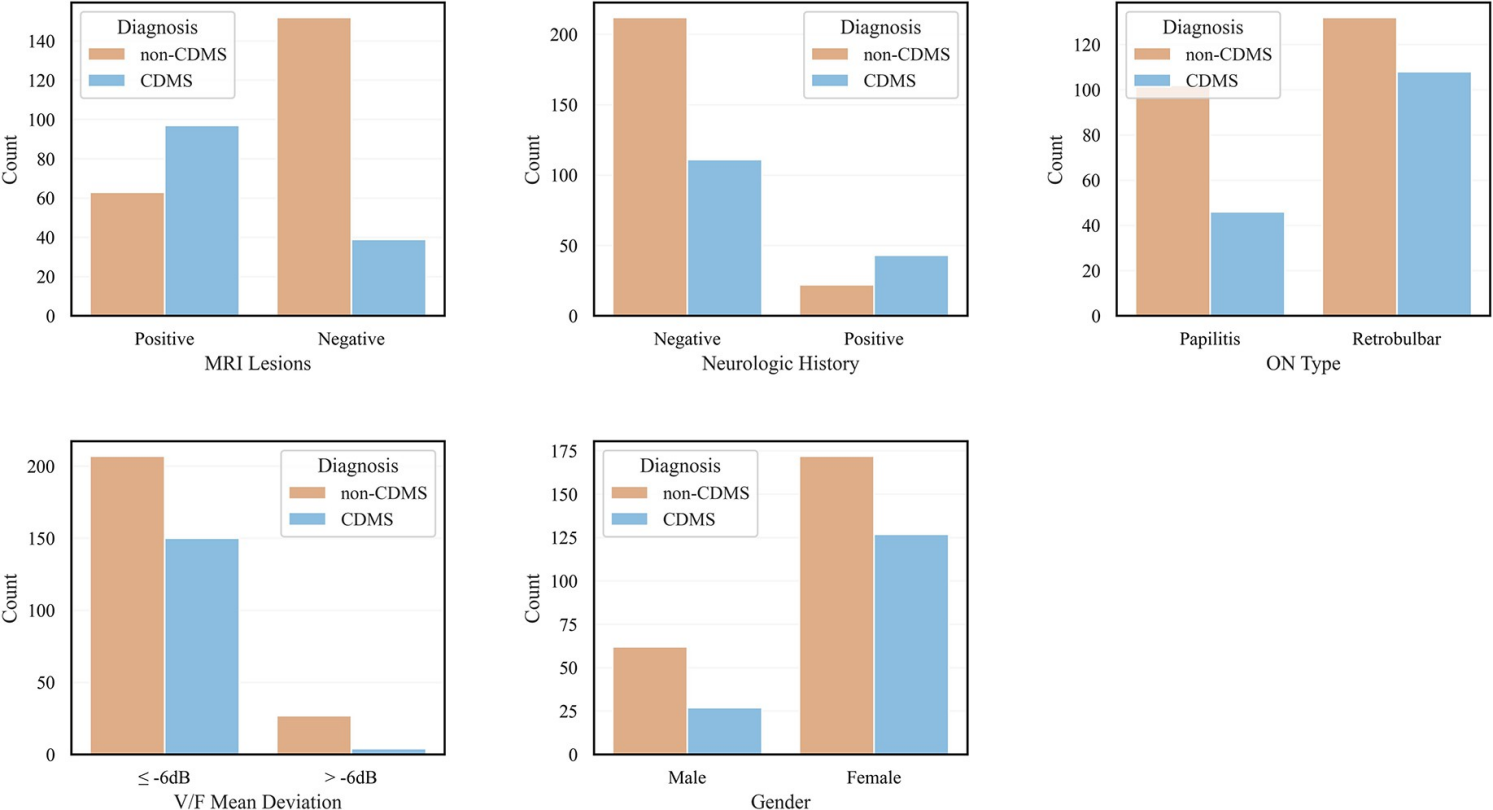
Bar plots indicating clinically definite multiple sclerosis (CDMS) and non-CDMS based on final predictors. MRI: Magnetic resonance imaging; ON: Optic neuritis; VF: Visual field.

In this study, we utilized the chi-squared test to assess the relationship between the categorical variables and either MS or non-MS diagnosis. We set a p-value threshold of 0.05 as the criterion for statistical significance. Specifically, variables with p-values less than or equal to this rounded threshold were deemed significant and were further examined in subsequent statistical tests. Five variables were statistically significant and were selected as the final features for the machine learning model, including MRI lesions, neurologic history, ON type, gender, and MD (Fig 3). Based on the rule of thumb that events per variable between 10 and 20 eliminate bias and provide reliable results [14,15], our sample size for this study was appropriate.

### Missing data

Two variables, MRI lesions, and MD, had missing values in 37 and 2 instances, respectively. To rectify this, we utilized the K-nearest neighbors (KNN) imputation method from the fancyimpute library [16]. This method estimates missing values by considering the information other variables provide in the dataset, excluding the target variable. The algorithm finds the KNN for the observation with missing data and then imputes it based on the non-missing elements in the neighborhood.

### Machine learning models

The analysis used Python version 3.10.9 and its associated libraries, namely Pandas, scikit-learn, and Matplotlib. We employed the decision tree (DT) classifier as our main model and trained it on the training data. The hyperparameters of the DT were manually tuned to achieve optimal performance on the cross-validation set. The model’s average performance across multiple metrics was computed and reported as the cross-validation results. Subsequently, the model was tested on the hold-out test set.

To ensure the robustness of our results and to provide a comparative analysis, we trained additional machine-learning models, including logistic regression (LR), random forest (RF), support vector machine (SVM) and multilayer perceptron (MLP). LR is a standard and routine method for the development of models that performs well in predicting disease risk [17]. RF is a machine learning model that builds an ensemble of decision trees and aggregates predictions [18]. RF can handle both classification and regression problems for categorical and numeric variables. The SVM is a machine learning model that classifies data by mapping inputs to a high-dimensional space and creating a linear decision boundary [19]. The MLP is an artificial neural network (ANN) that can learn and model complex relationships in data [20]. The performance of these models was evaluated on the cross-validation and hold-out test sets using the same metrics.

### Validation of models

We partitioned the data into two subsets to assess the model’s performance: a training set and a hold-out test set. The test set comprised 25% of the data, and we used a random state of 42 for reproducibility. During the training phase, we utilized the repeated stratified k-fold cross-validation technique, with a k value 5, to enhance robustness and minimize bias in the results [21,22]. This stratified data partitioning into five equal folds ensured that each fold maintained a similar proportion of classes as the entire dataset. We trained the model on four of these folds and evaluated it on the remaining one. This process was repeated five times, ensuring that each fold served as the test set once. This constitutes the 5-fold aspect of our cross-validation. The repetition implies that we performed this entire 5-fold process five times, each time with a different random division of the data. The final results of the cross-validation are the average of the results from all 25 folds (5 folds repeated five times).

### Class imbalance

In predictive modeling, class imbalance can significantly impact a model’s performance by biasing it towards the majority class. In our study, we quantified the degree of class imbalance using skewness. Initially, the dataset consisted of 179 non-CDMS cases and 112 CDMS cases, with a skewness of 0.42. To address this issue, we employed the synthetic minority oversampling technique (SMOTE) on the training set. SMOTE is a popular technique that generates synthetic samples from the minority class, thereby balancing the class distribution and improving model performance [23]. We used the imbalanced-learn library (version 0.12.3) with a sampling strategy of 0.9, k-neighbors set to 5, and a random state set to 42.

### Feature importance

We incorporated the SHapley Additive exPlanations (SHAP) library to calculate the feature importance. This library computes the average contribution of each variable to the prediction and provides their relative importance as a numerical value [24].

## Results

### Patients’ characteristics and development of multiple sclerosis

Our study included 388 patients, among whom 154 (40%) developed CDMS. The mean age of these patients was 31.7 ± 6.6 years. Of the 160 patients with at least one MRI lesion (41% of the total), 97 (61%) developed CDMS. Additionally, 20% of patients with normal MRI developed CDMS. Overall, 71% of patients who developed CDMS had an MRI lesion. Among the 65 patients with a positive neurologic history, 66% developed CDMS. Of the 299 female patients, 42.5% developed CDMS, compared to 30% of male patients. Among the 255 patients with a moderate to severe MD (≤ -6 dB), 42% developed CDMS, while 13% of patients with normal to mild MD converted to CDMS. Of the 240 patients with retrobulbar ON, 45% developed CDMS, compared to 31% with papillitis ON (Table 1 and Fig 1).

### Decision tree model

**Fig 4:**
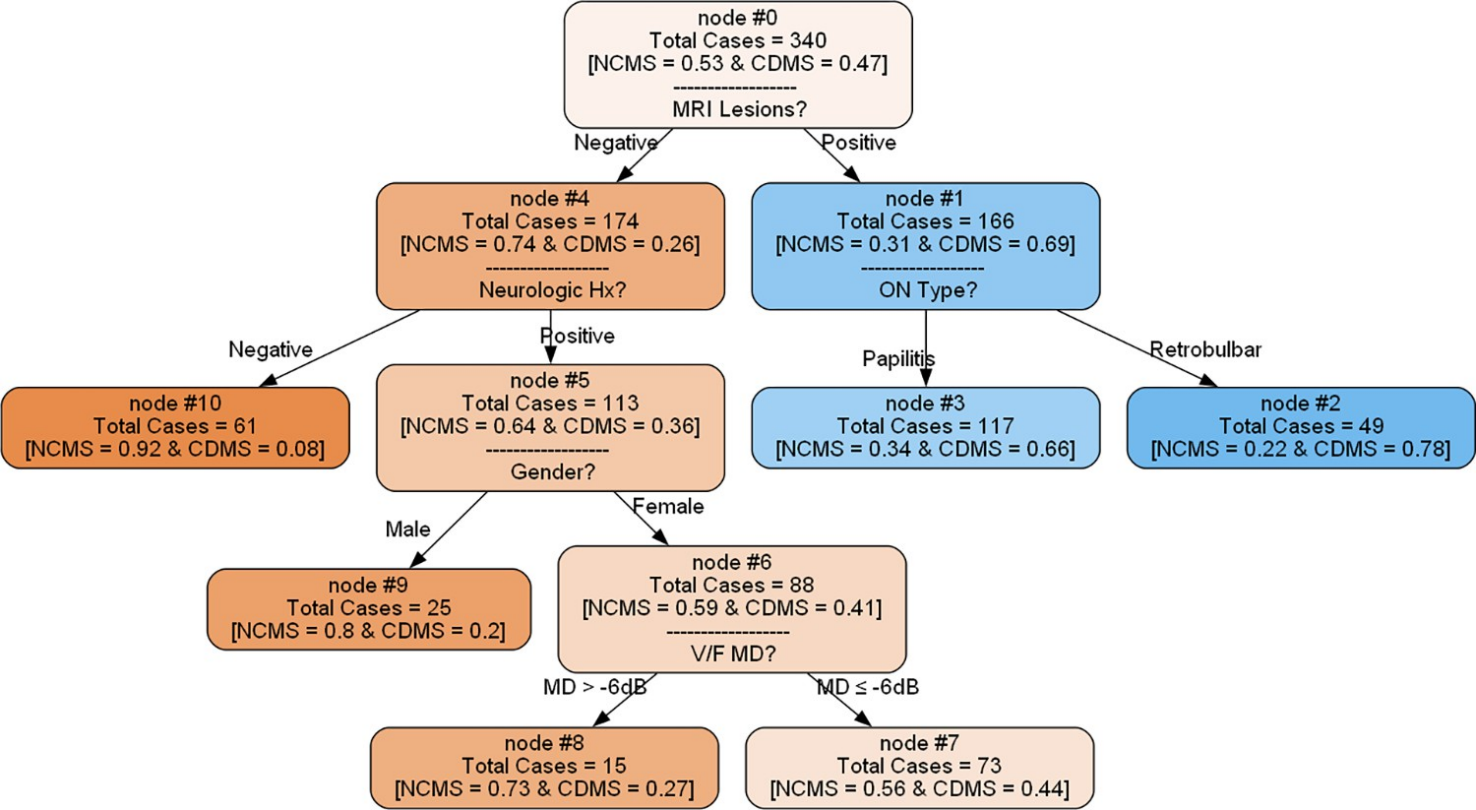
Decision tree models for predicting conversion of optic neuritis to multiple sclerosis. NCMS: None clinical multiple sclerosis; CDMS: Clinically definite multiple sclerosis; MRI: Magnetic resonance imaging; ON: Optic neuritis; Hx: History; VF: Visual field; MD: Mean deviation.

Applying SMOTE to the training data increased the number of CDMS cases to 161, resulting in a class distribution of 179 non-CDMS and 161 CDMS cases, and reduced skewness to 0.11. A decision tree was developed using a dataset of 340 training subjects, of which 47% eventually developed CDMS. The decision tree showed that 69% of patients with a positive MRI lesion converted to CDMS. The ON type added more detail to the prediction, with retrobulbar ON patients having a 78% conversion rate to CDMS. On the other hand, patients with a negative neurologic history had a lower conversion rate to CDMS, decreasing from the initial 26% at the root node to just 8%. Other factors, such as being male and having a MD greater than -6 dB, further indicated a lower chance of CDMS conversion (Fig 4).

### Models’ evaluation

**Fig 5:**
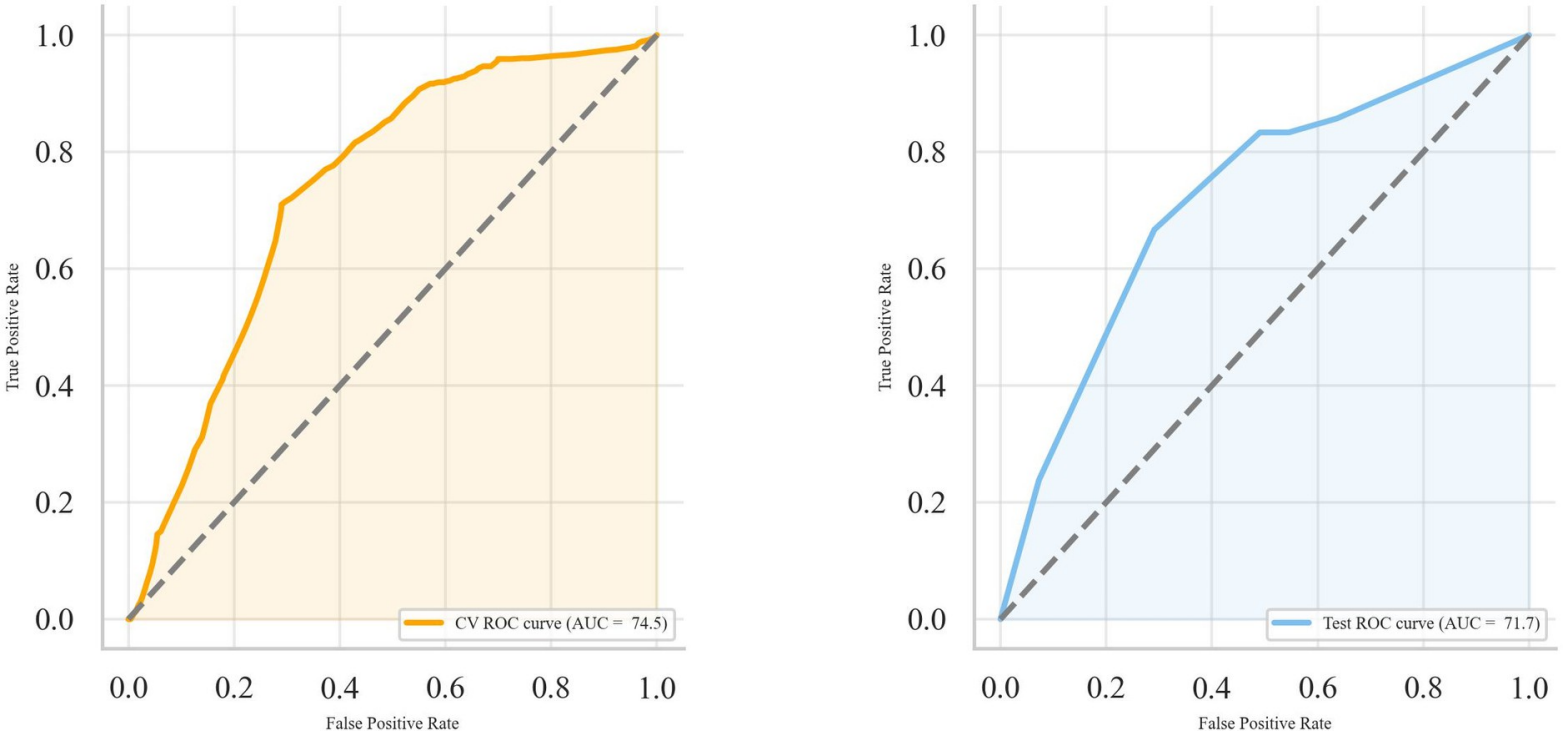
ROC curve of decision tree classifier on cross-validation (left) and test set (right).

We assessed the performance of DT model along with other models in predicting the risk of MS development in patients with ON using various metrics. The SVM model achieved the highest accuracy of 72.2% during cross-validation and 69.1% on the test set, with an F1-score of 71.3% and 65.1%, respectively. However, the DT model, with an accuracy of 71% and 69.1% in cross-validation and test set, an AUROC of 74.5% and 71.7%, respectively, provided the best balance of performance and interpretability (Table 2, Fig 5).

**Table 2:** Models’ performance metrics on the cross-validation and final test data.

Models	Accuracy	AUC	Specificity	Recall	PPV	NPV	F1 score
Decision Tree							
CV	71.0	74.5	71.1	70.9	69.1	73.1	69.9
Test	69.1	71.7	70.9	66.7	63.6	73.6	65.1
MLP							
CV	70.7	74.7	71.6	69.7	69.0	72.6	69.2
Test	69.1	71.3	69.1	69.0	63.0	74.5	65.9
Logistic Regression							
CV	69.2	75.1	72.7	65.2	68.4	70.1	66.6
Test	69.1	72.1	76.4	59.5	65.8	71.2	62.5
Random Forest							
CV	71.4	76.3	71.4	71.4	69.5	73.6	70.3
Test	69.1	70.5	70.9	66.7	63.6	73.6	65.1
SVM							
CV	72.2	76.0	71.5	72.9	70.0	74.7	71.3
Test	69.1	70.2	70.9	66.7	63.6	73.6	65.1

CV: Cross-validation, AUROC: Area under the receiver operating curve, PPV: Positive predictive value, NPV: Negative predictive value; MLP: Multilayer perceptron, SVM: Support vector machine.

**Fig 6:**
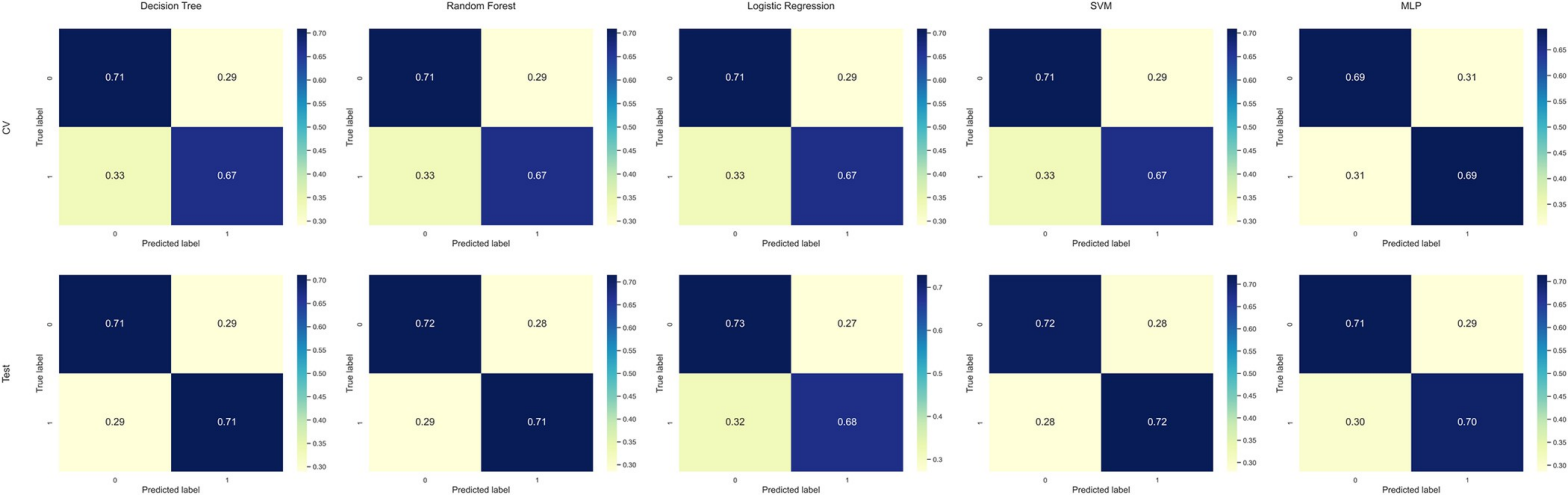
Confusion matrices of predictive models in the training and test dataset.

The MLP model achieved an accuracy of 70.7% during cross-validation and 69.1% on the test set, with AUC values of 74.7% and 71.3% for cross-validation and test sets, respectively. The F1 score was 69.2% for cross-validation and 65.9% for the test set. The LR model demonstrated an accuracy of 69.2% during cross-validation and 69.1% on the test set. The AUC values were 75.1% for cross-validation and 72.1% for the test set, with F1 scores of 66.6% and 62.5%, respectively. The RF model exhibited an accuracy of 71.4% during cross-validation and 69.1% on the test set, with corresponding AUC values of 76.3% and 70.5%. The F1 score was 70.3% for cross-validation and 65.1% for the test set. The SVM model yielded an accuracy of 72.2% during cross-validation and 69.1% on the test set, with AUC values of 76.0% and 70.2% for cross-validation and test sets, respectively. The F1 score was 71.3% for cross-validation and 65.1% for the test set. The Decision Tree’s confusion matrix shows a true positive rate of 67% and a true negative rate of 71% on the cross-validation set. On the test set, both the true positive and true negative rates are 71%. A comparative analysis of this with other models is depicted in Fig 6.

### Feature importance

**Fig 7:**
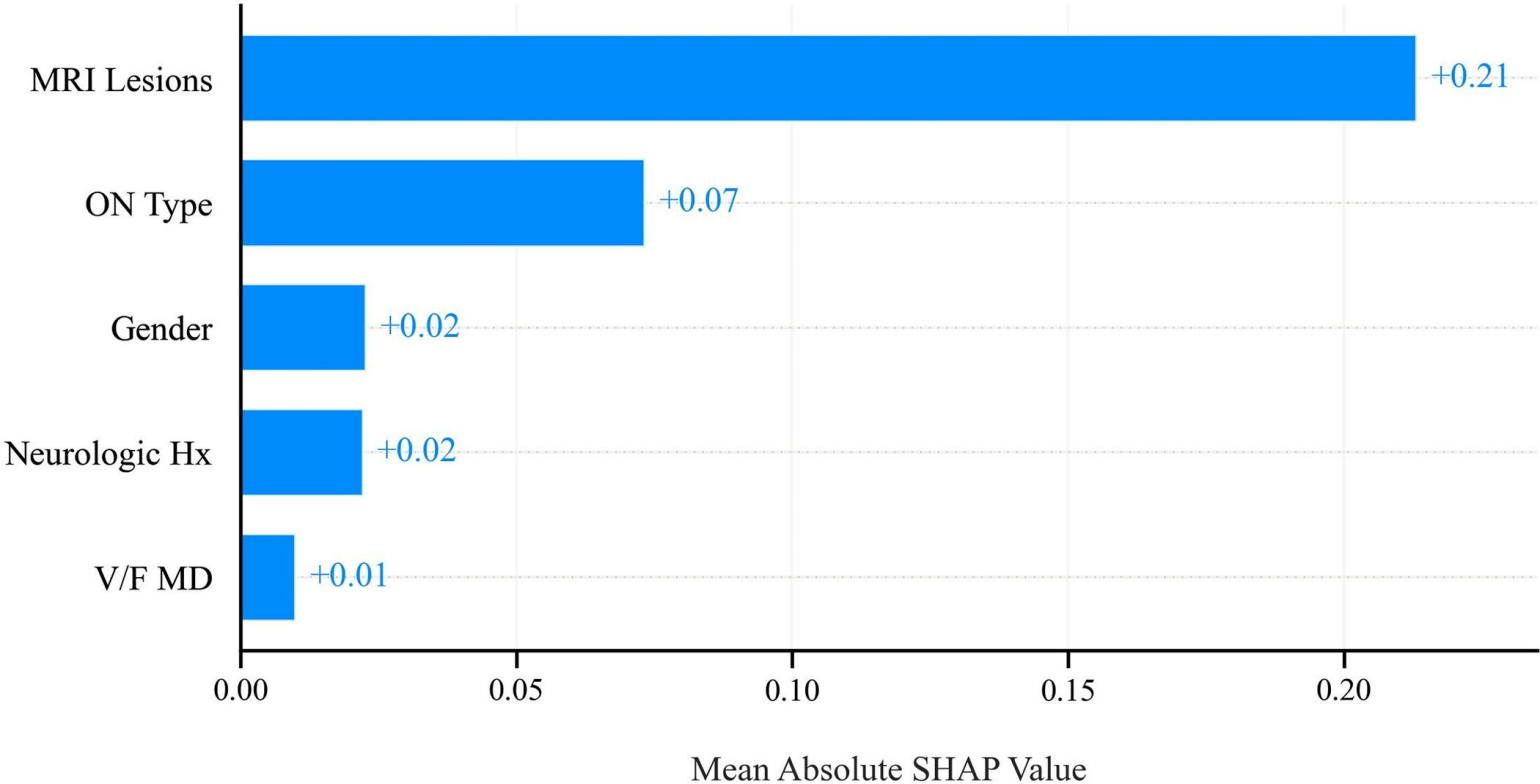
Feature importance of variables.

The SHAP analysis revealed that the most influential factor in predicting the conversion from ON to MS was the presence of MRI lesions, which had a mean absolute SHAP value of 0.21. This was followed by the type of ON, with a SHAP value of 0.07. Gender and history of neurological symptoms had similar impacts on the model’s prediction, both with SHAP values of 0.02. MD was the least impactful feature, with a SHAP value of 0.01 (Fig 7).

## Discussion

Machine learning has emerged as a powerful tool in the medical field, offering the potential to enhance diagnostic accuracy, predict disease progression, and personalize treatment plans. In the context of ON and its potential progression to MS, machine learning models can help identify patients at risk, thereby improving clinical care and aiding in decision-making for follow-up plans or the initiation of disease-modifying therapies [1,9]. In this study, we introduced a decision tree-based algorithm with practical applications in clinical settings, and implemented four other standard models, MLP, RF, LR, and SVM, to compare performance and evaluate the reliability of the DT model.

In our study, the SVM model demonstrated superior performance in terms of accuracy and F1-score but the differences were not substantial enough to outweigh the practical advantages of the DT model (Table 2). The complexity and lack of interpretability of SVM model limit its immediate applicability in clinical settings. Hence, we focused on the decision tree algorithm due to its enhanced interpretability, making it closer to physicians’ decision-making process [25]. Machine learning methods like DT are appropriate for analyzing data within a predetermined set of categories, especially when input variables are distinct, and the final classification is binary [26]. Explanation and interpretation are challenging aspects of ML and prediction models [27], and most models require a web-based tool [9,28,29]. DT model makes subgroups in the data, stratifies risk within each group and is easy to interpret for physicians, making it suitable for clinical use [30].

Patients with ON have different baseline findings at the onset that can be valuable for predicting the conversion to MS. Previous studies have highlighted this relationship [31–34]. These studies conducted univariate regression, followed by multivariate logistic regression, leading to decreased accuracy [35]. A few studies have applied comprehensive models and machine learning algorithms to predict the conversion of ON to MS (Table 3). Luo et al. [9] introduced a prognostic model for assessing the risk of this conversion in comparison to using MRI plaque as a single predictor. They concluded that the prognostic model performed better than routine practice. Abri-Aghdam et. al [6] used four variables in Iranian patients, including gender, type of ON, history of ON, and plaque in MRI, to construct a prediction model for ON conversion to MS; they achieved an accuracy of 74%, a sensitivity of 71%, a specificity of 76%, as well as a positive predictive value (PPV) and negative predictive value (NPV) of 65% and 79%, respectively.

**Table 3:** Comparison of previous predictive models.

Study	Method	Sample size	Group	Number of predictors	Accuracy	AUROC	Web-based
Present study	Decision tree	388	ON	5	70%	74.9%	No
Luo et al. [9]	Cox proportional hazards	388	ON	5	72%	77%	Yes
Abri et al. [6]	Decision tree	356	ON	4	74%	-	No
Rasouli et al. [11]	Extreme Gradient Boosting (XGBoost)	273	CIS (ON + transverse myelitis)	9	78.3%	85.8%	Yes
Olesen et al. [10]	Logistic regression	40	ON	3	-	89%	No

ON: Optic neuritis; CIS: Clinically isolated syndrome; AUROC: Area under the receiving curve.

The performance of these models is comparable to our model, which achieved an accuracy of 70% and an AUROC of 74.5%. The differences in performance metrics are not substantial, indicating that our model is competitive with existing models. Our study utilized five statistically significant variables to create a comprehensive model based on these findings. With an NPV of 73%, our model effectively identifies patients less likely to develop MS, allowing physicians to plan appropriate follow-ups. Since PPV and NPV are related to the condition’s prevalence, further testing and validation in subsequent studies are necessary to confirm these findings. However, our model provides sufficient evidence for its utility in clinical settings.

In contrast, the model developed by Olesen et al. [10] utilized biomarkers from cerebrospinal fluid (CSF). Despite achieving higher performance metrics, the invasiveness of CSF biomarker collection contrasts with the non-invasive nature of our predictive model, which relies solely on clinical and imaging data. This invasiveness may limit the practical application of their model in routine clinical settings despite its higher performance metrics.

The differences in model performance across various studies can be attributed to several factors: Variations in sample size and baseline characteristics, including age, gender distribution, and clinical profiles, significantly impact model performance. Models with higher complexity, such as Extreme Gradient Boosting (XGBoost) or deep learning techniques, often achieve better performance metrics but can be less interpretable and more prone to overfitting. Moreover, our study used statistically significant features, but other studies may have included different variables that enhanced their models’ predictive power.

Our findings align with previous studies that have identified factors contributing to the conversion to MS, as shown by the feature importance calculated using the SHAP library (Fig 5). According to previous studies, gender, neurologic symptoms, ON type, and MRI lesions were significant predictors [4,6,34]. Notably, our study revealed that MD has a key role in predicting conversion of ON to MS, and ON patients with mild MD had a lower chance of developing MS. DT modeling revealed that lesions on MRI have a 69% probability of converting to MS in ON patients. The probability of developing MS is 26% in the absence of MRI plaque (Fig 4). Thus, it is essential to stratify risk in patients with no evidence of MS at baseline MRI. When there are no MRI plaques, the next step is to consider the patient’s history of neurologic symptoms and gender to understand the patient’s risk profile better. Finally, MD provides more discrimination to patients. Female patients with mild MD and a positive history of neurologic symptoms but no lesion at MRI have a 27% chance of developing MS, while those with moderate-severe MD have a 44% chance.

A strength of our study is our use of the ONTT database, which still remains a reliable study in this field due to the large sample size, comprehensive data, and long-term follow-up. Leveraging this data, we introduced an algorithmic model for more informed decision-making by physicians. To reduce the risk of overfitting, characterized by a model having significantly higher performance on training data compared to testing data, we employed a method known as repeated stratified k-fold cross-validation. By implementing this approach, we aimed to obtain more reliable estimates of the model’s performance that consider variations due to data splitting and randomization. This technique enabled us to assess the model’s generalization capability and stability in predicting the target variable based on the selected features and it is another strength of the present study. However, our study is not without limitations. The use of Poser criteria for diagnosing MS may not fully capture the complexity and variability of the disease. Furthermore, we lacked access to the actual MRI images, limiting our imaging data to the information available in the study reports and dataset. If we had these images, we could utilize deep learning models specifically designed for MRI data analysis. These models are capable of extracting complex patterns from the images.

## Conclusion

Our decision tree model, exhibiting satisfactory performance, effectively stratifies patients based on baseline findings. The information provided by this model will assist physicians in making informed decisions regarding each patient’s future condition. In addition, while our model achieved similar predictive accuracy to other standard models such as MLP, RF, LR, and SVM, it is important to note that these models may offer different advantages depending on the specific clinical context and available resources. For instance, while our decision tree model offers enhanced interpretability, other models may be more suitable for handling larger datasets or more complex feature interactions. Our study underscores the potential of machine learning in enhancing clinical decision-making and patient care in the context of ON and MS.

We recommend future studies with larger sample sizes and comprehensive data collection to validate or refine our model. In particular, incorporating additional patient characteristics and clinical variables or combination with deep learning models could further improve the model’s predictive accuracy. Moreover, exploring other machine learning techniques or ensemble methods could also provide valuable insights into predicting ON conversion to MS.

## References

[1] Kale N. Optic neuritis as an early sign of multiple sclerosis. Eye Brain. 2016;8:195–202. Epub 20161026. doi: 10.2147/EB.S54131 ; PubMed Central PMCID: PMC5398757. 28539814 PMC5398757

[2] Kazim SF, Islam M, Khan M, Hameed B, Shafqat S. Risk of multiple sclerosis after idiopathic optic neuritis in a Pakistani population. Can J Neurol Sci. 2010;37(2):258–63. doi: 10.1017/s0317167100010027 . 20437939

[3] Bennett JL. Optic Neuritis. Continuum (Minneap Minn). 2019;25(5):1236–64. doi: 10.1212/CON.0000000000000768 ; PubMed Central PMCID: PMC7395663. 31584536 PMC7395663

[4] Multiple sclerosis risk after optic neuritis: final optic neuritis treatment trial follow-up. Arch Neurol. 2008;65(6):727–32. doi: 10.1001/archneur.65.6.727 ; PubMed Central PMCID: PMC2440583. 18541792 PMC2440583

[5] Sørensen TL, Frederiksen JL, Brønnum-Hansen H, Petersen HC. Optic neuritis as onset manifestation of multiple sclerosis: a nationwide, long-term survey. Neurology. 1999;53(3):473–8. doi: 10.1212/wnl.53.3.473 . 10449106

[6] Abri Aghdam K, Aghajani A, Kanani F, Soltan Sanjari M, Chaibakhsh S, Shirvaniyan F, et al. A novel decision tree approach to predict the probability of conversion to multiple sclerosis in Iranian patients with optic neuritis. Mult Scler Relat Disord. 2021;47:102658. Epub 20201128. doi: 10.1016/j.msard.2020.102658 . 33279796

[7] Beck RW, Trobe JD, Moke PS, Gal RL, Xing D, Bhatti MT, et al. High- and low-risk profiles for the development of multiple sclerosis within 10 years after optic neuritis: experience of the optic neuritis treatment trial. Arch Ophthalmol. 2003;121(7):944–9. doi: 10.1001/archopht.121.7.944 . 12860795

[8] Tintore M, Rovira À, Río J, Otero-Romero S, Arrambide G, Tur C, et al. Defining high, medium and low impact prognostic factors for developing multiple sclerosis. Brain. 2015;138(Pt 7):1863–74. Epub 20150421. doi: 10.1093/brain/awv105 . 25902415

[9] Luo W, Deng X, Xu X, Song R, Luo M, Moss HE, et al. Development of a Prognostic Model for Predicting Multiple Sclerosis After Optic Neuritis: A Secondary Analysis of Data From the Optic Neuritis Treatment Trial. J Neuroophthalmol. 2022;42(1):88–96. Epub 20211022. doi: 10.1097/WNO.0000000000001424 ; PubMed Central PMCID: PMC9159903. 34860745 PMC9159903

[10] Olesen MN, Soelberg K, Debrabant B, Nilsson AC, Lillevang ST, Grauslund J, et al. Cerebrospinal fluid biomarkers for predicting development of multiple sclerosis in acute optic neuritis: a population-based prospective cohort study. J Neuroinflammation. 2019;16(1):59. Epub 20190311. doi: 10.1186/s12974-019-1440-5 ; PubMed Central PMCID: PMC6410527. 30857557 PMC6410527

[11] Rasouli S, Dakkali MS, Azarbad R, Ghazvini A, Asani M, Mirzaasgari Z, et al. Predicting the conversion from clinically isolated syndrome to multiple sclerosis: An explainable machine learning approach. Mult Scler Relat Disord. 2024;86:105614. Epub 20240409. doi: 10.1016/j.msard.2024.105614 . 38642495

[12] Beck RW, Cleary PA, Anderson MM Jr, Keltner JL, Shults WT, Kaufman DI, et al. A randomized, controlled trial of corticosteroids in the treatment of acute optic neuritis. The Optic Neuritis Study Group. N Engl J Med. 1992;326(9):581–8. doi: 10.1056/NEJM199202273260901 . 1734247

[13] The clinical profile of optic neuritis. Experience of the Optic Neuritis Treatment Trial. Optic Neuritis Study Group. Arch Ophthalmol. 1991;109(12):1673–8. doi: 10.1001/archopht.1991.01080120057025 . 1841573

[14] Ogundimu EO, Altman DG, Collins GS. Adequate sample size for developing prediction models is not simply related to events per variable. J Clin Epidemiol. 2016;76:175–82. Epub 20160308. doi: 10.1016/j.jclinepi.2016.02.031 ; PubMed Central PMCID: PMC5045274. 26964707 PMC5045274

[15] Vittinghoff E, McCulloch CE. Relaxing the rule of ten events per variable in logistic and Cox regression. Am J Epidemiol. 2007;165(6):710–8. Epub 20061220. doi: 10.1093/aje/kwk052 . 17182981

[16] fancyimpute: An Imputation Library for Python [Internet]. 2016. Available from: https://github.com/iskandr/fancyimpute.

[17] Nusinovici S, Tham YC, Chak Yan MY, Wei Ting DS, Li J, Sabanayagam C, et al. Logistic regression was as good as machine learning for predicting major chronic diseases. J Clin Epidemiol. 2020;122:56–69. Epub 20200310. doi: 10.1016/j.jclinepi.2020.03.002 . 32169597

[18] Huang Y, Huang Z, Yang Q, Jin H, Xu T, Fu Y, et al. Predicting mild cognitive impairment among Chinese older adults: a longitudinal study based on long short-term memory networks and machine learning. Front Aging Neurosci. 2023;15:1283243. Epub 20231023. doi: 10.3389/fnagi.2023.1283243 ; PubMed Central PMCID: PMC10626462. 37937119 PMC10626462

[19] Cortes C, Vapnik V. Support-vector networks. Machine Learning. 1995;20(3):273–97. doi: 10.1007/BF00994018

[20] Murtagh F. Multilayer perceptrons for classification and regression. Neurocomputing. 1991;2(5–6):183–97.

[21] Pedregosa F, Varoquaux G, Gramfort A, Michel V, Thirion B, Grisel O, et al. Scikit-learn: Machine learning in Python. the Journal of machine Learning research. 2011;12:2825–30.

[22] Refaeilzadeh P, Tang L, Liu H. Cross-Validation. In: Liu L, ÖZsu MT, editors. Encyclopedia of Database Systems. Boston, MA: Springer US; 2009. p. 532–8.

[23] Chawla NV, Bowyer KW, Hall LO, Kegelmeyer WP. SMOTE: synthetic minority over-sampling technique. Journal of artificial intelligence research. 2002;16:321–57.

[24] Lundberg S, Lee S-I. A Unified Approach to Interpreting Model Predictions 2017.

[25] Henrard S, Speybroeck N, Hermans C. Classification and regression tree analysis vs. multivariable linear and logistic regression methods as statistical tools for studying haemophilia. Haemophilia. 2015;21(6):715–22. Epub 20150807. doi: 10.1111/hae.12778 . 26248714

[26] Song YY, Lu Y. Decision tree methods: applications for classification and prediction. Shanghai Arch Psychiatry. 2015;27(2):130–5. doi: 10.11919/j.issn.1002-0829.215044 ; PubMed Central PMCID: PMC4466856. 26120265 PMC4466856

[27] Marcinkevičs R, Vogt JE. Interpretable and explainable machine learning: A methods-centric overview with concrete examples. WIREs Data Mining and Knowledge Discovery. 2023;13(3):e1493. doi: 10.1002/widm.1493

[28] Veloso M. A web-based decision support tool for prognosis simulation in multiple sclerosis. Mult Scler Relat Disord. 2014;3(5):575–83. Epub 20140502. doi: 10.1016/j.msard.2014.04.005 . 26265269

[29] Rasouli S, Dakkali MS, Azarbad R, Ghazvini A, Asani M, Mirzaasgari Z, et al. Predicting the Conversion from Clinically Isolated Syndrome to Multiple Sclerosis: An Explainable Machine Learning Approach. Multiple Sclerosis and Related Disorders. doi: 10.1016/j.msard.2024.105614 38642495

[30] Cruz JA, Wishart DS. Applications of machine learning in cancer prediction and prognosis. Cancer Inform. 2007;2:59–77. Epub 20070211. ; PubMed Central PMCID: PMC2675494. 19458758 PMC2675494

[31] Morrow SA, Fraser JA, Nicolle D, Kremenchutzky M. Predicting conversion to MS—the role of a history suggestive of demyelination. Can J Neurol Sci. 2010;37(4):488–91. doi: 10.1017/s0317167100010519 . 20724257

[32] Falcão-Gonçalves AB, Bichuetti DB, de Oliveira EML. Recurrent Optic Neuritis as the Initial Symptom in Demyelinating Diseases. J Clin Neurol. 2018;14(3):351–8. Epub 20180531. doi: 10.3988/jcn.2018.14.3.351 ; PubMed Central PMCID: PMC6031992. 29856159 PMC6031992

[33] Marques IB, Matias F, Silva ED, Cunha L, Sousa L. Risk of multiple sclerosis after optic neuritis in patients with normal baseline brain MRI. J Clin Neurosci. 2014;21(4):583–6. Epub 20130823. doi: 10.1016/j.jocn.2013.06.013 . 24231563

[34] Ro LS, Yang CC, Lyu RK, Lin KP, Tsai TC, Lu SR, et al. A prospective, observational study on conversion of clinically isolated syndrome to multiple sclerosis during 4-year period (MS NEO study) in Taiwan. PLoS One. 2019;14(7):e0202453. Epub 20190715. doi: 10.1371/journal.pone.0202453 ; PubMed Central PMCID: PMC6629056. 31306415 PMC6629056

[35] Xing F, Luo R, Liu M, Zhou Z, Xiang Z, Duan X. A New Random Forest Algorithm-Based Prediction Model of Post-operative Mortality in Geriatric Patients With Hip Fractures. Front Med (Lausanne). 2022;9:829977. Epub 20220511. doi: 10.3389/fmed.2022.829977 ; PubMed Central PMCID: PMC9130605. 35646950 PMC9130605

